# Towards pixel-wise area-at-risk characterization with cardiac BOLD MRI at rest

**DOI:** 10.1186/1532-429X-18-S1-P63

**Published:** 2016-01-27

**Authors:** Marco Bevilacqua, Rohan Dharmakumar, Sotirios Tsaftaris

**Affiliations:** 1 grid.4305.20000000419367988The University of Edinburgh, Edinburgh, UK; 2 grid.462365.00000000417909464IMT Institute of Advanced Studies, Lucca, Italy; 3 grid.412041.2000000012106639XUniversity of Bordeaux, Talence, France; 4 grid.50956.3f0000000121529905Biomedical Imaging Research Institute, Cedars-Sinai Medical, Los Angeles, CA USA

## Background

Characterizing area-at-risk has become a sought-after clinical indicator for the treatment of cardiovascular disease. MRI can provide such information without contrast and radiation. Recently, cardiac phase-resolved blood-oxygen-level-dependent (CP-BOLD) MRI, has shown that this is possible at rest, without even stress agents. However, most of the necessary post-processing analysis remains segmental and does not take advantage of the full spectrum of available information within the acquired data.

We hypothesize that pattern recognition methods can assess the likelihood of myocardial ischemia considering time series of myocardial signal intensity and motion synergistically.

## Methods

Subjects: Flow and motion-compensated 2D short-axis CP-BOLD were acquired at 1.5T, mid ventricle, in canines (n = 8) at baseline and under severe LAD stenosis, held for 3 hours. During reperfusion, LGE images were acquired. Image analysis: For each CP-BOLD acquisition, myocardial contours were traced and segmented in 36 radially consecutive segments in a semi-automated fashion, providing a time series for each segment. A time series for wall motion was obtained as the within segment average radial distance between endo- and epi-cardial boundaries across the cardiac cycle. The algorithm iteratively decides if a time series deviates from a linear decomposition model. At each iteration, a time series not conforming to the model is found using a one-class-support-vector-machine and a new model is learned (with dictionary learning). This process leads to a per-segment classification (and confidence value): remote or ischemic. We then define ischemic extent (IE) as the relative fraction of the myocardium that is ischemic. IE is derived also with the S/D approach (ratio of intensity systole vs diastole) with ICA (an unsupervised independent component analysis decomposition). From LGE images relative infarct size is measured. Statistical analysis: Correlations between IE and infarct size are determined for each method.

## Results

The figure shows an example of an ischemia likelihood map derived with our proposed method. Observe the close correspondence between the derived map and LGE, capturing a broad ischemic territory (and potential area-at-risk), which eventually led to the diffuse endocardial infarct. By averaging likelihoods within a segment we can see a 6-segment bulls-eye representation.

The table summarizes together with bootstrapped p-values (after 10^6^ permutations), how well area-at-risk measured with different methods correlates with infarct size. The proposed method using both intensity and wall motion and optimal thresholds yields optimal performance (correlation of 0.84 with infarct size). Even using only intensity it outperforms all others (p = 0.04). All other methods yield non-significant correlations.

## Conclusions

We found that unsupervised determination of ischemic myocardium that is vulnerable to infarction can be accurately assessed. This approach remains to be tested in patients.

**Table 1:** Pearson correlation coefficients r and related p-values, obtained by correlating, for different methods, ischemic extent (IE) with infarct size.

Method	r	bootstrapped p-value
S/D	-0.08	0.57
ICA	0.51	0.26
Similar to the proposed using only intensity and fixed threshold	0.32	0.22
Proposed method using only Intensity	0.66	0.04
Proposed method using both intensity and wall motion	0.84	0.004

**Figure 1:**
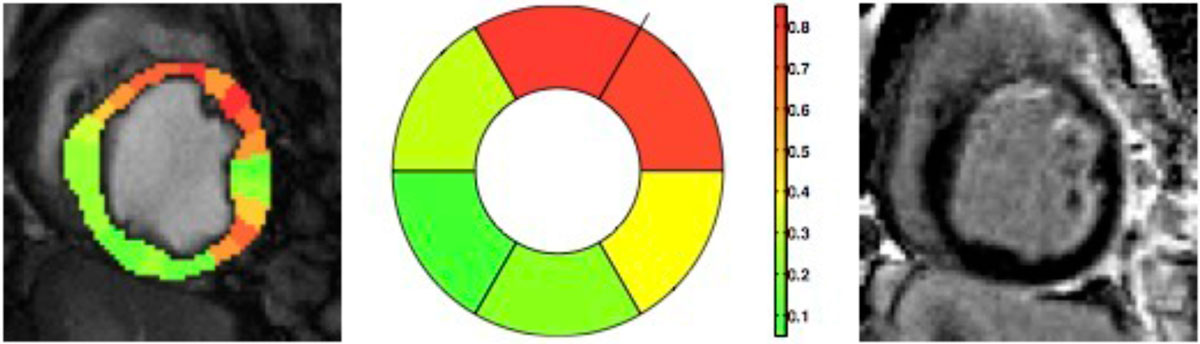
**An ischemia likelihood map color-coded and overlaid on the original CP--BOLD image in diastole (left)**. Together also shown a 6-segment bulls-eye plot of likelihood (middle); the color bar shown refers to both. Corresponding LGE image (right).

